# Circular RNA in hepatocellular carcinoma: emerging therapeutic strategies

**DOI:** 10.3389/fimmu.2025.1722576

**Published:** 2025-12-09

**Authors:** Gregor Jazbec, Tadeja Režen

**Affiliations:** Centre for Functional Genomics and Bio-Chips, Institute of Biochemistry and Molecular Genetics, Faculty of Medicine, University of Ljubljana, Ljubljana, Slovenia

**Keywords:** circRNA, RNA-based therapeutics, hepatocellular carcinoma, immunotherapy, circRNA-based therapeutics

## Abstract

Circular RNAs, or circRNAs, are unique single-stranded RNA molecules that form covalently closed circles. They possess a wide range of cellular functions and can act as tumor suppressors or promoters, regulating various aspects of carcinogenesis. These functions include acting as microRNA sponges, interacting with and binding to proteins and mRNA, regulating transcription and RNA splicing, translating into proteins, and serving as cargo in exosomes. Due to their diverse cellular roles and enhanced molecular stability, circRNAs are not only promising biomarkers but also emerging candidates for the development of RNA-based therapeutics. In this review, we explored the therapeutic potential of circular RNA by reviewing the current state of the art in the field of oncology, with a particular focus on hepatocellular carcinoma. Based on their endogenous functions, circular RNA can be designed, synthesized and delivered to produce various proteins, such as neoantigens and cytokines, or to sequester oncogenic microRNA in tumor cells. Although many therapeutic applications of circular RNA are still in the early stages of development, they have already shown promising results in both *in vitro* and *in vivo* animal models. In summary, circular RNAs are emerging as both therapeutics and biomarkers in liver cancer and broader oncology, with relevant clinical applicability, particularly in immunotherapy.

## 1 Introduction

Liver cancer represents a substantial global health challenge, ranking as the sixth most prevalent cancer and the third leading cause of cancer-related deaths worldwide (second in men), with its incidence steadily increasing (1). Hepatocellular carcinoma (HCC) accounts for over 80-90% of liver cancer cases. The etiology of HCC is diverse, with the main primary risk factors being chronic hepatitis B and C, alcohol consumption, and metabolic disorders such as non-alcoholic fatty liver disease (NAFLD) or metabolic dysfunction-associated fatty liver disease (MAFLD). Epidemiological data indicate a shift in etiological risks from viral to non-viral factors (2). Liver cancer has a low overall five-year survival rate of 18.8% worldwide (3), and a recurrence rate of up to 70% (4). Although surgical resection, transplantation, or local ablation are options for early-stage patients, approximately 50% of HCC patients ultimately require systemic treatment. Immunotherapies have advanced rapidly in recent years (5). Among these, therapies based on immune checkpoint inhibitors have become established as first-line treatment for advanced HCC. However, approximately two-thirds of patients do not respond to these therapies, indicating that a significant proportion of patients with advanced HCC cannot benefit from existing treatment approaches (5).

In recent years, understanding of the immune system’s involvement in HCC has increased. Different etiologies of HCC influence the immune response and result in unique tumor immune microenvironment features, dictating distinct approaches to immunotherapy (5). HCC tumors have been classified into two major classes, inflamed and non-inflamed, according to immune-based stratification, with several subclasses: active, exhausted, immune-like, intermediate, and excluded (6). The development of precision therapies in HCC is challenging due to significant inter-patient heterogeneity, a limited number of druggable cancer drivers, and difficulties in establishing patient-derived models (7, 8). Numerous intrinsic and extrinsic factors contribute substantially to the remarkable diversity observed in patients with HCC. Moreover, clinicopathological factors, rather than genetic alterations, have been identified as the major determinants of outcomes for patients with advanced HCC treated with immune checkpoint inhibitors. Future studies should explore alternative biomarkers such as the level of immune activation or the pretreatment composition of the immune tumor microenvironment (9). However, these discoveries have not yet been translated into improved diagnosis or prediction of patient response to available immunotherapies. Therefore, there is a pressing need to identify biomarkers for treatment response and to develop new therapies for resistant cases.

CircRNAs or circular RNA are extensively studied in HCC due to their significant clinical potential. Numerous studies have revealed the molecular functions of circRNAs in HCC tumors, including their roles in immune response and response to immunotherapy (10–13). Many studies have investigated the biomarker potential of circRNAs in the plasma of HCC patients. A recent meta-analysis of 82 studies, including a total of 15,024 patients, confirmed that circRNAs in combination with mRNAs-specifically hsa_circ_000224, hsa_circ_0003998, KIAA0101 mRNA, and GPC-3 (Glypican 3) mRNA-are optimal diagnostic liquid biopsy-based biomarkers for HCC (14). However, none of these discoveries have yet been introduced into clinical practice. Here, we review recent developments in the field of circRNAs in HCC as potential RNA-based therapeutics. The exploitation of endogenous circRNA functions and the generation of synthetic circRNAs can be applied in various therapeutic approaches in oncology; however, most are still in pre-clinical testing and have shown limited translation to HCC. As the field of RNA-based therapeutics advances and our understanding of factors influencing immunotherapy response improves, we expect that new circRNA-based therapeutics will be introduced into clinical practice.

## 2 Biogenesis and classification of eukaryotic circRNA

Eukaryotic circRNAs are generated from precursor mRNA transcribed by RNA polymerase II (15). In a two-step process, the immature pre-mRNA undergoes non-canonical back-splicing, regulated by spliceosomal machinery and intronic complementary sequences (ICSs) (16–18). In this reaction the 2’-OH group of the RNA adenine nucleotide attacks the 5’ splice site, generating a lariat intron loop at the 3’ end of the exon and a free 3’-OH group at the 5’ end (19). The lariat, which can also form its own circular RNA (20, 21), is subsequently removed through a cascade of reactions, allowing the two remaining exon ends to join and form a covalently closed circRNA (19). To date, multiple models of circRNA loop formation based on circRNA composition have been described (22), including intron pairing-driven (23, 24), RNA-binding protein (RBP)-driven (25, 26) and variants of lariat-driven circularization (20, 27–29).

Depending on their genomic origin and biogenesis pathways, circRNAs are mainly classified into three distinct groups: exonic circRNAs (ecircRNAs), derived from single or multiple exons of pre-mRNA; exonic-intronic circRNAs (EIciRNAs), which contain both exonic and intronic sequences; and circular intronic RNAs (ciRNAs), derived from introns through spliceosome-mediated splicing (18, 20, 23). Most ciRNAs and EIciRNAs are retained in the nucleus, whereas ecircRNAs are predominantly transported to the cytoplasm by the nuclear export system.

CircRNAs are dysregulated in various cancers, including HCC and are present in body fluids, making them good biomarkers (30). Their lack of 5’ caps and 3’ poly(A) tails significantly increases their resistance to degradation by RNA exonucleases, allowing them to accumulate in specific cell types even though back-splicing is generally less efficient than linear splicing (16, 31).

## 3 Molecular functions of circRNA and their links to disease

Initially, circular RNAs were considered non-functional by-products of transcription (32); however, various mechanisms and cellular functions have since been demonstrated for circRNA. Importantly, these functions and their mechanisms have been directly linked to the development and progression of HCC. They are also associated with drug resistance in various malignancies, including HCC (30). In this chapter, we briefly review the functions of circRNA in HCC.

### 3.1 Competing endogenous RNA: the miRNA sponges

Historically, the most studied and defined function associated with circRNA is their role as competing endogenous RNA (ceRNA) (33). The binding of miRNAs by circRNA increases transcription rates of miRNA-targeted mRNA. CircRNA can act as a tumor suppressor by binding an oncogenic miRNA or as an oncogene by binding a tumor suppressor miRNA. Both mechanisms have been described in HCC. For example, circASAP1 was identified as a prognostic predictor in HCC, and acts as an oncogene by binding tumor suppressors miRNA, miR-326 and miR-532-5p, which regulate the MAPK1 and CSF-1 pathways (34). Another example in HCC is circMTO1, which acts as a tumor suppressor by binding oncogenic miR-9 and promoting p21 expression (35). Intratumoral administration of cholesterol-conjugated siRNA targeting circMTO1 promoted tumor growth in mice *in vivo*. In HCC the involvement of exosomal circRNAs has been demonstrated multiple times, either as tumor suppressors, promoters, or regulators of treatment resistance. Exosomes enriched with hsa_circ_0000563, an oncogenic circRNA, facilitated tumor growth by binding miR-148a-3p and regulating MTF-1 (metal-regulatory transcription factor-1) (36). Exosomal hsa_circ_0051443 is significantly downregulated during HCC and acts as a tumor suppressor by upregulating BAK1 (BCL2 Antagonist/Killer 1) as a ceRNA for miR-331-3p, thereby promoting apoptosis and arresting the cell cycle (37). By binding miRNA, circRNA can also affect immune cells and tumor response to immunotherapy in HCC. Exosomal circUHRF1 inhibited NK (natural killer) cell function by binding miR-449c-5p (10). This upregulated TIM-3 (T cell immunoglobulin and mucin domain 3) and promoted immune evasion and resistance to anti-PD1 immunotherapy. CircCCAR1 also promoted resistance to anti-PD1 immunotherapy by binding miR-127-5p and upregulating WTAP (Wilms tumor 1-associated protein) (12). Exosomes containing circCCAR1 are taken up by CD8 + T cells, where they facilitate dysfunction of these cells by stabilizing PD-1 (Programmed Cell Death 1). Circ_HMGCS1 promotes chemoresistance by binding miR-338-5p and upregulating IL-7 expression (13).

### 3.2 Binding of proteins: affecting their stability and function

The unique circRNA sequences and tertiary structures enable them to bind various proteins, affecting protein function and degradation. Numerous oncogenic and tumor suppressor roles, including regulation of responses to immune therapies, have been identified in HCC. CircIPO11 recruits DNA TOP1 (topoisomerase 1) to the *GLI1* (GLI Family Zinc Finger 1) promoter, initiating its transcription (38). This activates the sonic hedgehog signal transduction cascade and promotes self-renewal of cancer cells. Administration of ASO (antisense oligonucleotides) against circIPO11 near the tumor in mice inhibited tumor growth *in vivo*. CircPSD3 inhibited vascular invasion and metastasis in HCC by binding HDAC1 (histone deacetylase 1), preventing its translocation to the nucleus and thus its function (39). CircCDYL binds and stabilizes HRNR (hornerin) protein and enhances immunotherapy resistance in HCC by upregulating of mTORC1-p70S6K signaling and PD-L1 expression (11).

CircRNA can also function as scaffolds. CircCCNY, or hsa_circ_0000235, enhances HCC sensitivity to Lenvatinib and suppresses immune evasion by serving as a scaffold for binding HSP60 and the E3 ubiquitin ligase SMURF1 (SMAD Specific E3 Ubiquitin Protein Ligase 1), leading to degradation and inactivation of the MAPK signaling pathway (40). Circ-ADD3 inhibits HCC metastasis by enhancing the interaction between CDK1 (Cyclin Dependent Kinase 1) and the oncoprotein EZH2 (Enhancer of Zeste 2 Polycomb Repressive Complex 2 Subunit), resulting in EZH2 ubiquitination and degradation in HCC (41). CircIPP2A2 also serves as a molecular scaffold in modulating the Hornerin/PI3K/AKT/GSK3β axis and promotes HCC tumorigenesis (42). CircATF6 acts as a scaffold to form a circATF6/CALR/CAPN2 ternary complex, facilitating degradation of CALR (Calreticulin) by CAPN2 (Calpain 2), thereby inhibiting HCC progression (43).

Several circRNAs have been found to bind various RNA-binding proteins and thereby affect their function. CircDLC1 binds HuR (or ELAVL1 – ELAV like RNA binding protein 1), blocks its interaction with *MMP1* (matrix metallopeptidase 1) mRNA, and facilitates mRNA degradation (44). This significantly decreases tumor size in mice *in vivo*. CircPABPN1 reduces *PABPN1* (Poly(A) Binding Protein Nuclear 1) mRNA translation by sequestering HuR (45). The oncogenic hsa_circ_00074854 binds HuR and enhances HuR protein stability (46). We have found that hsa_circ_0062682 binds YBX1 (Y-box-binding protein 1) and promotes oncogenesis in HCC cell lines (47). Oncogenic circCPSF6 binds PCPB2 (Poly(RC) Binding Protein 2) and prevents degradation of *YAP1* (Yes1 Associated Transcriptional Regulator) mRNA (48).

CircRNA can also have dual roles, binding both proteins and RNA or expressing peptides. CircMALAT1 (hsa_circ_0002082) forms a complex with ribosomes and the mRNA of the tumor suppressor gene *PAX5* (paired box 5), preventing its translation and thereby promoting self-renewal of HCC stem cells (49). In the same study, circMALAT1 was also found to bind miR-6887-3p and upregulate the JAK2 pathway. CircSTX6 was found to bind the HNRNPD (Heterogeneous Nuclear Ribonucleoprotein D) protein, thereby facilitating its binding to *ATF3* mRNA, and to encode a polypeptide circSTX6-144aa, via IRES1 (Internal ribosome entry site 1) (50). Both functions of this circRNA independently promoted HCC progression.

### 3.3 Translation of circRNA

Some circRNAs can associate with ribosomes and be translated cap-independently via embedded IRES (51, 52). In addition, N^6^-methyladenosine (m^6^A) modifications of RNA promote efficient protein translation from circRNA in human cells (53). Several peptides or proteins encoded by circRNA have already been identified in various cancers, including HCC (54). One example is the β-catenin-370aa protein translated from circ-0004194, which promotes HCC malignancy through the Wnt/β-catenin pathway by acting as bait for GSK3β (Glycogen Synthase Kinase 3 Beta), which is responsible for β-catenin ubiquitination and degradation with the ubiquitin ligase β-TrCP (Beta-Transducin Repeat Containing E3 Ubiquitin Protein Ligase) (55). Another example is circZKSAN1, which translates into circZKSaa and interacts with the tumor suppressor FBXW7 (F-Box And WD Repeat Domain Containing 7). This facilitates mTOR ubiquitination and subsequent suppression of HCC by inhibiting the PI3K/AKT/mTOR pathway (56). Recently, the protein circPETH-147aa was found to remodel the immunosuppressive microenvironment in HCC (57). CircPETH is transported via extracellular vesicles from tumor-associated macrophages to HCC cells, where it is translated via IRES and leads to metabolic oncogenic reprogramming of the cells.

In summary, the primary functions of circRNA - mediated through RNA and protein binding, as well as translation into peptides - highlight their potential applications in developing emerging circRNA-based therapies for HCC. CircRNA can act as protein scaffolds, influence protein function, and affect mRNA expression by sequestering both miRNA and RBP. They can also produce peptides with various functions. Furthermore, circRNA can have dual roles as sponges and in protein production. Studies of endogenous circRNA in HCC already suggest several applications in RNA-based therapies. Further research into circRNA functions in different cancers is needed to elucidate their various roles and to design synthetic circRNA that could be applied in therapy.

## 4 Therapeutic applications of synthetic circRNA in HCC

**Figure 1:**
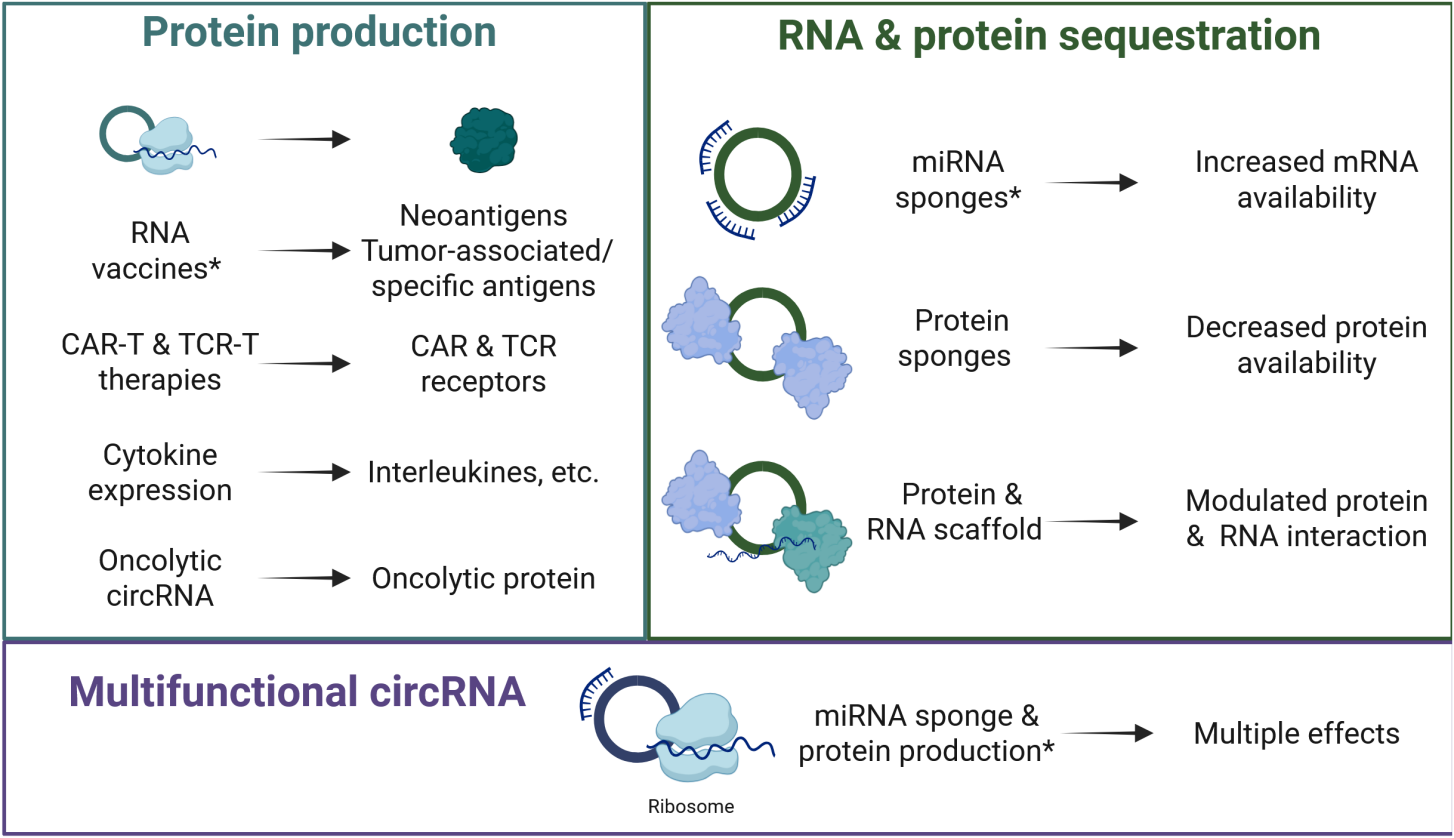
CircRNA-based therapeutics can be developed for protein production, RNA and protein sequestration, and with multifunctional roles. *CircRNA-based therapies in preclinical testing for HCC. Created in BioRender. CAR-T, chimeric antigen receptor T cells; circRNA, circular RNA; TCR-T, transgenic T cell receptor.

The endogenous functions of circRNAs and their reported roles in disease have been identified as potential targets for various therapies. In addition to endogenous circRNAs, recent advances in RNA synthesis have enabled the development of synthetic circRNAs with therapeutic potential (**Figure 1**). These synthetic circRNAs are delivered to their targets through different mechanisms such as lipid nanoparticles or viral vectors and have specific functions in modulating disease progression. They include circRNA-based anti-cancer treatments that have either already demonstrated effectiveness or are currently in development for different types of cancer. We present the current published circRNA-based treatments and their therapeutic applications with examples in HCC (**Table 1**).

**Table 1:** Brief overview of published circRNA-based hepatocellular carcinoma therapies.

Therapy type	Products	Functionality	Delivery method	Successful application	Reference
RNA vaccine	mutated PTPN2 neoantigen	induces host dendritic cell maturation and T-cell activation	LNPs	*in vitro* Hepa1–6 cell line, *in vivo* C57/B6 mice	(61)
RNA vaccine	GPC3 antigens	Enhances antigen presentation and strengthens the interaction between cDC1 and CD8^+^T cells	LNP	*in vitro* Hepa1–6 and hepatoma-22 cell lines, *in vivo* in subcutaneous and orthotopic HCC model and NRAS/c-Myc-driven model in C57BL/6J and BALB/c mice	(62)
miRNA sequestration		sequesters hepatitis C miRNA miR-122	electroporation	*in vitro* HuH-7.5 cell line	(63)
miRNA sequestration		sequesters miRNAs that target tumor suppressors ANGPTL1, SOCS3, ACACB, and EHHADH	LNPs	*in vitro* THLE-3 HepG2, HCCLM3, PLC, and Huh-7 cell lines*, in vivo* NOD/SCID mice	(64)
miRNA sequestration + protein expression	anti-PD-1 single-chain variable fragment	sequesters oncogenic miR-25 and enhances immune response	LNPs	*in vitro* Hepa1–6 cell line*, in vivo* C57BL/6 mice	(65)

ACACB, Acetyl-CoA Carboxylase Beta; ANGPTL1, Angiopoietin Like 1; EHHADH, Enoyl-CoA Hydratase And 3-Hydroxyacyl CoA Dehydrogenase; GPC3, Glypican 3; HCC – hepatocellular carcinoma; LNPs – lipid nanoparticles; PD-1, Programmed Cell Death 1; PTPN2, Protein Tyrosine Phosphatase Non-Receptor Type 2; SOCS3, Suppressor Of Cytokine Signaling 3.

### 4.1 Protein production

The most common therapeutic application of synthetic circRNAs is their ability to express specific proteins, with different applications depending on the protein expressed. Properly purified circRNAs exhibit low immunogenicity and significantly greater stability, providing prolonged and persistent expression over time compared to their linear mRNA counterparts (58–60). Additionally, the absence of a translation re-initiation step in rolling circle replication allows for more efficient translation (51), requiring less RNA and making delivery less toxic (66). However, this approach requires some additional modification of the circRNA, such as inserting self-cleaving peptides to produce functional proteins (67). Under certain stress conditions, such as viral infections, cap-dependent mRNA translation is inhibited, while IRES- or m6A- dependent translation of circRNAs is not (68), further increasing their relative efficiency. Several potential applications of circRNA-based protein production have already been demonstrated successfully.

#### 4.1.1 RNA vaccines

CircRNA vaccines have been a hot topic of discussion since their successful use against COVID-19. During that time, circRNA was synthesized *in vitro* and delivered *in vivo* to express SARS-CoV-2 antigens, such as its binding domain, to trigger the host immune response (58). However, viral antigens are not the only products that vaccines can express. Tumor cells often express T lymphocyte surface checkpoint inhibitors and evade the immune response (69). This, combined with their innate low immunogenicity, enables cancer cells to escape immune detection. Neoantigens are non-autologous tumor proteins generated by genomic mutations, aberrant RNA splicing, abnormal post-translational modifications, or the incorporation of viral ORFs (open reading frame). They have strong immunogenicity and are not expressed in normal tissues (70, 71), making them good selective targets for cancer therapy. By overexpressing these tumor-specific neoantigens encoded on synthetic circRNAs, it is possible to significantly increase the host immune response. This was already demonstrated for different mRNAs (72, 73) and for a circRNA, circMYH9, which expressed immunogenic neoantigens in patient-derived organoids of colorectal cancer (74). TAAs (tumor-associated antigens) or TSAs (tumor-specific antigens) can also be selected as targets for expression in addition to neoantigens, as demonstrated *in vivo* in mouse models treating melanoma by inducing chicken ovalbumin expression from circRNA (75). However, TAAs often trigger poor T-cell responses largely due to immune tolerance. The canonical role of immune tolerance is to prevent harmful autoimmune responses by removing T-cells that react to self-antigens. Combining multiple shared TAAs has recently become more common. The selected TAAs are often widely expressed in related tumors and can induce antitumor immune responses when combined with different vectors or adjuvants (76).

Another approach is to combine vaccine therapies with PD-1 monoclonal antibodies, which inhibit the binding of PD-1 on T-cells to PDL-1, thereby preventing immune escape of tumor cells (77). PD-1 treatment usually has low response rates. However, when combined with mRNA vaccines, these response rates increase and offer a potential new approach to developing therapies (78–82).

The design of components for the circRNA platform used in RNA vaccines production, such as optimal IRES sites (83), and circRNA delivery mechanisms, including novel lipid nanoparticles (LNPs) and exosomes (84, 85), is advancing significantly but still faces challenges such as low delivery rates and biocompatibility (86). According to vaccine assessment reports on COVID-19 vaccines, up to 21% of LNPs were detected in the liver during preclinical trials, regardless of the initial injection site or method of injection. This may lead to off-target expression and tissue damage, highlighting the need for improvements in delivery specificity (87). One emerging method for non-liver tissues is the inclusion of miR-122 binding sites within UTRs (untranslated regions) of therapeutic RNA product. This approach significantly reduces therapeutic expression when the RNA is incorrectly localized to the liver *in vivo* in mice (88).

CircRNA vaccines targeting HCC, like most other RNA-based tumor vaccines, have not yet reached clinical trials. However, their development is progressing using various expressed proteins. One group designed a circRNA neoantigen vaccine expressing a polypeptide. The circRNA was designed to express a self-splicing intron encoding mutated *PTPN2* (Protein Tyrosine Phosphatase Non-Receptor Type 2) and was delivered via a circRNA-lipid nanoparticle complex both *in vitro* and *in vivo* in mice (**Table 1**) (61). The vaccine significantly inhibited tumor growth in a dose-dependent manner in a subcutaneous murine HCC model. In this model, the vaccine did not cause any obvious toxic side effects or significant impact on liver function. In the subcutaneous murine HCC tumor model, the vaccine also eradicated tumors and induced both innate and adaptive immune responses. Furthermore, the vaccine had a prophylactic effect in Hepa1–6 tumor-bearing mice. They also confirmed *in vitro* that the circRNA system is more stable and longer-lasting than linear RNA and expresses higher levels of peptides.

**Figure 2:**
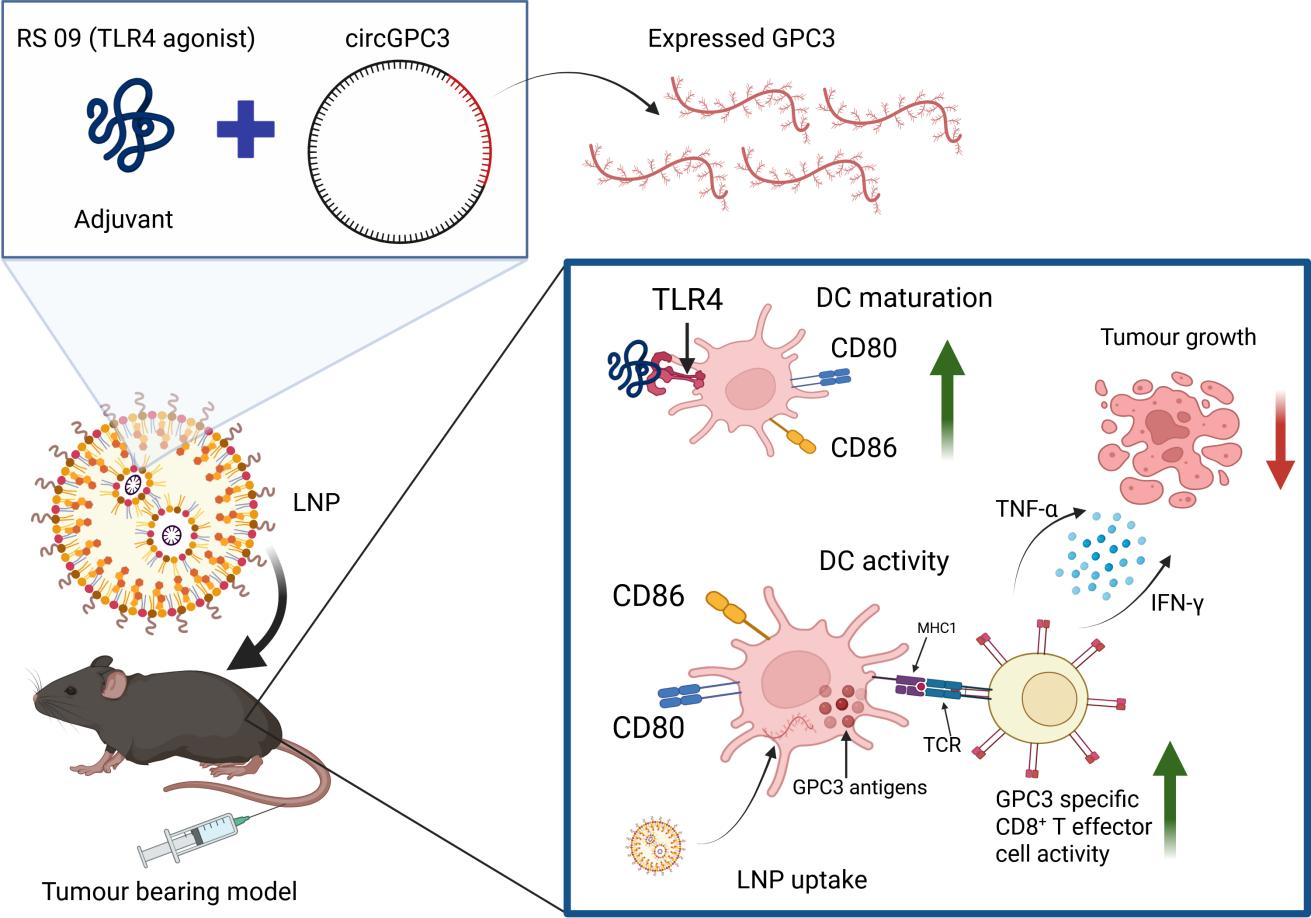
A circRNA-based vaccine encoding GPC3 is in preclinical testing for hepatocellular carcinoma (62). CircGPC3 was administered with a TLR4 agonist as an adjuvant to enhance the immune response. The vaccine plus adjuvant slowed tumor growth in different mouse tumor models, increased tumor infiltration by immune cells, and elevated inflammatory cytokine levels. Created in BioRender. DC, dendritic cells; GPC3- glypican 3; IFN-γ, interferon-γ; LNP, lipid nanoparticle; MHC1, major histocompatibility complex 1; TCR, T cell receptor; TLR4, Toll-like receptor-IV; TNF-α, tumor necrosis factor alpha.

Another group has successfully designed a circRNA-based vaccine encoding *GPC3* for HCC (**Table 1**) (62). GPC3 is highly expressed in HCC and has already been used in developing CAR-T therapies for treating HCC as well as tested in a phase I clinical trial for safety and antitumor activity (89–91). The circGPC3 vaccine demonstrated sustained antigen production, overcoming the inherent limitations of traditional mRNA vaccines, and elicited a durable antitumor immune response (62). The group also added a TLR4 (Toll-like receptor-IV) agonist as an adjuvant to further enhance immune responses by disrupting immune tolerance in HCC (**Figure 2**). In combination with the TLR4 agonist, the circGPC3 vaccine effectively suppressed tumor progression in subcutaneous and orthotopic tumor mouse models, and showed a favorable safety profile with no apparent evidence of tissue damage (62). In hepa1–6 cells, this circRNA outperformed mRNA in translation efficiency and expression stability.

Development of circRNA vaccines in HCC is a very promising area, and studies of gene expression in HCC tumors have already revealed new potential antigens such as *AURKA* (Aurora Kinase A) (92) and neoantigens (74) that could be considered for the design of novel circRNA vaccines.

#### 4.1.2 CAR-T and TCR-T therapies

Among the most advanced immunotherapeutic approaches are CAR-T (chimeric antigen receptor T cells) and TCR-T (transgenic T cell receptors). CAR-T cell therapy involves extracting patient’s T lymphocytes and genetically modifying them *in vitro* to express CARs. This modification enables the T cells to recognize, bind to, and eliminate tumor cells. Traditional CAR-T treatments are costly and time-consuming, as the cells are modified either using viral (lentiviral or γ-retroviral) vectors (93, 94) or CRISPR-mediated gene delivery (95), both of which carry risks of genome integration. CAR-T therapies still face translational barriers, including the need for lymphodepletion, CRS (cytokine release syndrome), and neurotoxicity (96). Persistent CAR-T cell activity also induces long-term B cell aplasia, significantly impairing the reconstitution of humoral immunity (97). Some *in vivo* CAR-T therapies have already entered clinical trials; however, those with circRNA cargo have not yet done so. They are expected to enter trials in the next few years, but so far they have been developed for the treatment of multiple myeloma, B cell cancers and autoimmune diseases (91).

Treatment of solid tumors remains challenging due to antigen loss. This occurs when tumors develop that no longer express the targeted antigen during treatment, often as a results of factors such as inflammation-induced dedifferentiation in melanomas (98). Tumor heterogeneity, where not all tumor cells express the antigen targeted by CAR-T cells, can also pose a problem (99). Another common issue is the phenomenon known as “on-target off-tumor” toxicity (100). This occurs when therapeutic CAR cells attack non-malignant tissues that express the target antigen, resulting in damage to normal tissues. If the target antigen is expressed on immune cells, this can also lead to the killing of proximal immune cells. Considering these toxicities, selecting appropriate target antigens is critical for expanding the applicability of CAR-based therapies. However, identifying such antigens has proven challenging, as most tumor antigens are also present on normal tissues (100). CAR-T therapy versions based on circRNA could potentially avoid some of these drawbacks, such as toxicity.

Studies have shown that some of these barriers can be alleviated by combining CAR-T treatments with mRNA vaccine boosts that enhance cells to produce increased quantities of IFN-γ, activating more T cells and eliciting a stronger immune response in mice and patients, respectively (99, 101). Alternative effector cell types are also being considered, notably NK cells. NK cells offer several potential advantages over T cells, including a more favorable cytokine profile. Furthermore, their non- MHC (major histocompatibility complex)-restricted cytotoxicity circumvents the need for patient-specific manufacturing. Numerous clinical studies have investigated the potential of NK cells and produced promising results further prompting their consideration as effectors (100, 102, 103). In addition, a group developed an *in vivo* panCAR platform that utilizes immune cell-tropic LNPs to deliver CAR-encoding circRNAs, thereby generating *in vivo* panCAR functional effector cells, including CAR-T, CAR-NK cells, and CAR-macrophages, demonstrating potent anti-tumor activity in preclinical models (104). The biggest obstacle in developing circRNA CAR therapies is not the use of circRNA over other expression vectors but rather the common shortcomings of CAR therapies.

TCR-T therapy is similar to CAR-T therapy, as it also involves extraction and genetic modification of the patient’s T cells (105). Unlike CAR-T cells, which bind to cell surface antigens, TCR-T cells recognize both surface and, importantly, intracellular proteins presented by major histocompatibility complex (MHC) molecules on the cell surface (106). This allows for a broader range of antigen recognition than CAR-T therapies and greater sensitivity to antigens. Earlier studies have shown that even a single peptide-MHC complex on target cells can trigger T cell activation (107, 108). The potential efficacy of TCR-T cells has been demonstrated in phase I clinical trial in HBV (hepatitis B virus)-related HCC patients (109) and in other solid tumors such as melanoma, synovial sarcoma, and myeloma, among others (110). Generation of TCR-T cells is mostly achieved through viral vectors or CRISPR editing. However, in a recent study, Shen et al. developed circular mRNA delivered by electroporation to produce TCR-T cells expressing the human cytomegalovirus (CMV) pp65 antigen, with killing effects observed *in vitro* and *in vivo* in a CDX mouse model (111). Both CAR-T and TCR-T anti-tumor therapies using mRNA have been tested in HCC models. Known CAR-T target for the treatment of HCC include Glypican 3 (GPC3) (89, 90), CD133 (112, 113), hepatocyte growth factor receptor (HGFR) (114), NKG2D (115), CD147 (116), alpha fetoprotein (AFP) (117), C-X-C Motif Chemokine Receptor 2 (CXCR2) (118), and CD39 (119, 120). Some have already been tested in clinical trials, each with significant limitations and specific drawbacks (121, 122). Although a TCR-T circRNA model in HCC was used to treat patients with HBV-related HCC (109), this remains a poorly explored topic. In addition, none of the above peptides have been expressed from circRNA vectors, which presents opportunities to construct and test new, potentially more efficient therapeutics.

Using circular RNA in CAR-T and TCR-T therapies is an emerging field, and these approaches are entering clinical trials for various diseases. However, so far, only one TCR-T therapy has been tested for HCC. Although circRNA-based platforms are being developed for other solid cancers, they could potentially be used for treatment of HCC in the future.

#### 4.1.3 Cytokine expression

Perhaps the simplest approach for protein expression and subsequent tumor environment modulation is the direct intratumoral injection of circRNA encoding anti-tumor cytokines. Often, antigens are incapable of activating T cells (123), and many trigger ineffective T-cell responses due to factors such as T-cell tolerance (124). Therefore, expressing cytokines that act as vaccine adjuvants, such as interferons, TNF (Tumor Necrosis Factor), IL-12 (interleukin 12), IL-2 (interleukin 2), and IL-6 (interleukin 6), can offer an alternative for treating antigen- or otherwise treatment-resistant tumors. This therapeutic approach faces several limitations. Cytokines such as IL-2 usually do not specifically activate CTLs (cytotoxic lymphocytes) in the tumor and are taken-up by many peripheral cells, resulting in a relatively short half-life (125). Cytokines also often bind with higher affinity to high-affinity receptors, which are constitutively expressed on off-target cells like Tregs, than to the low-affinity receptors expressed on T cells and NK cells. This is why higher doses of cytokines must be administered to achieve a CTL-mediated immune response to the tumor, leading to severe toxicity in most patients. Delivery specificity and tumor penetration also remain issues, as with other similar therapeutic methods (125, 126).

By using circRNA, most of these problems can be avoided due to higher and more durable *in vivo* protein expression (58). Several studies have demonstrated promising results using this approach in different cancers, both *in vitro* and *in vivo*, in mouse models. Yang et al. produced circRNA encoding four different cytokines: active IL15 (interleukin 15), a fusion protein of IL15 and the sushi domain of IL15Rα; IL12 single chain (IL12sc), a fusion protein of p35 and p40; GM-CSF (granulocyte-macrophage colony-stimulating factor); and IFNα 2b (interferon alpha 2b) (127). Intratumoral administration of the circRNA mixture resulted in significant regression of B16F10 syngeneic melanoma tumor growth in C57BL/6 mouse models, demonstrating that circRNA-directed local expression of cytokines in the tumor can suppress its growth and increase tumor infiltration of cytotoxic CD8^+^ T cells (127). Other groups have also successfully tested a similar approach in syngeneic mouse tumor models and humanized human tumor mouse models (128), and have applied their own modifications to improve efficiency, such as immunomodulatory ursodeoxycholic acid lipid nanoparticles (129). Like other RNA-based treatments, this approach is still in its early stages, and few examples are available. Currently, there are no cases of successful use in HCC (except as an adjuvant to a miRNA sequestration platform described later (65)). However, since cytokines like IL-2 have been shown to play a pivotal role in HCC (130–133), it is important to highlight this opportunity for the development of therapies in which such cytokines could be continuously overexpressed or stimulated by a circRNA platform.

### 4.2 RNA and protein sequestration

The widely studied endogenous function of circRNAs as miRNA sponges, sequestering miRNAs in a sequence-dependent manner to inactivate their function, was quickly considered for the development of synthetic therapeutics. In theory, this mechanism can be redirected to any miRNA target by designing circRNAs with specific miRNA-binding sites. Their circular structure confers resistance to exonucleolytic decay pathways activated during miRNA binding. As miRNAs are often expressed in various pathological processes (134, 135), they become targets for circRNA therapies that can rescue cells from aberrant phenotypes. For the design of circRNAs with miRNA sequestration function, the introduction and optimization of homologous MBSs (miRNA binding sites) in linear RNA precursors is essential (136). MBSs are designed to match the mature sequences of targeted miRNAs; for the assembly strategy, the MBS elements in linear RNA precursors of circRNAs are advised to contain mismatches rather than perfect antisense sequences (137). Perfectly base-paired sequences can form more stable interactions with miRNAs and would be vulnerable to AGO2 (Argonaute RISC Catalytic Component 2)-mediated endonucleolytic cleavage despite their circular nature (138).

In addition to sequestering miRNAs, circRNA-based therapies can also be used to sequester proteins. For example, artificial circRNAs with CA-repeat or CA-rich sequence clusters can efficiently and specifically modulate splicing by acting as sponges that bind hnRNPL (heterogeneous nuclear ribonucleoprotein L), a protein responsible for alternative splicing in mammals (139). The bound circRNA effectively deactivates the protein and its transport between the nucleus and cytoplasm in cell models. This has promising applications in hnRNPL-mediated genetic diseases. By engineering the sponge-type circRNA platform, the equilibrium between circRNAs and other interacting molecules can be disrupted to achieve therapeutic or regulatory effects on gene expression (139). Many RBPs could serve as potential targets of circRNAs, such as an oncogenic FXR1 (fragile X-related protein 1). Its dysregulation is linked to multiple cancer types, including HCC, and is emerging as a critical RBP involved in tumorigenesis through its interactions with target mRNAs (140, 141). This highlights the potential for further unexplored applications of circRNAs as tools for sequestration of carcinogenic RBPs.

**Figure 3:**
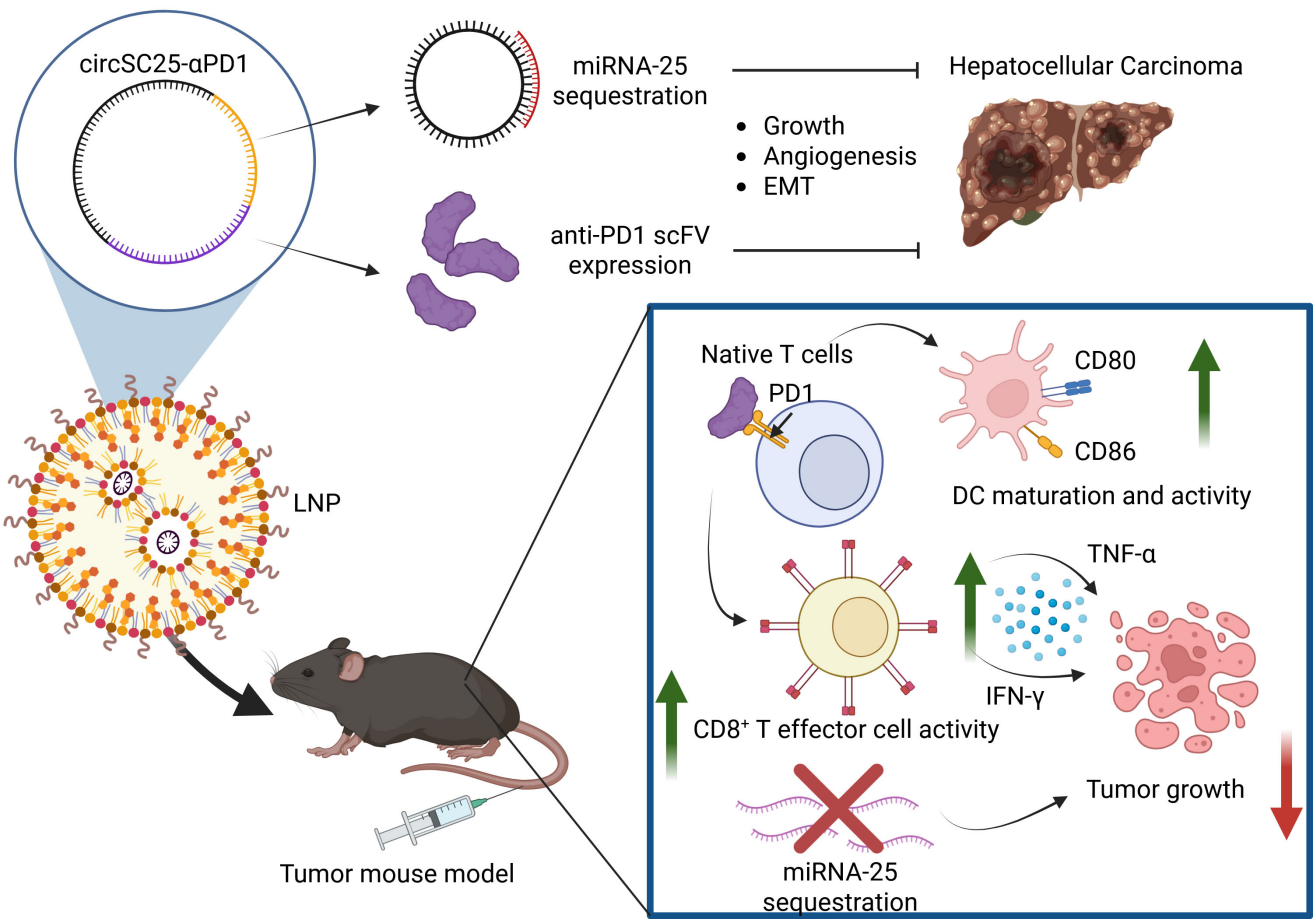
A circRNA-based therapy is in preclinical testing for hepatocellular carcinoma using a multifunctional artificial circRNA (65). CircSC25-αPD1 acts as a sponge for oncogenic miR-25 and also expresses an anti-PD-1 single-chain variable fragment (scFv). This circRNA demonstrated potent tumor-suppressive effects by inhibiting tumor growth *in vivo* in both subcutaneous and orthotopic tumor mouse models injected with LNP-circSC25-αPD1 via tail vein. The synthetic circRNA inhibited EMT and tumor angiogenesis, enhanced T cell proliferation, and increased immune response. Created in BioRender. DC, dendritic cells; EMT, epithelial–mesenchymal transition; IFN-γ, Interferon-γ; LNP, lipid nanoparticle; PD-1, Programmed Cell Death 1; TNF-α, Tumor necrosis factor alpha.

Due to the high variability in circRNA expression and the resulting large volume of research published over the years. HCC is one of the diseases in which the endogenous functions of circRNA in cancer have been extensively studied. Several studies on HCC have also demonstrated the therapeutic potential of synthesized circRNA sponge platforms. A well-known earlier example is the use of artificial circRNA to sequester hepatitis C miRNA miR-122, which impedes HCV (hepatitis C virus) progression *in vitro* in a HCC cell line (**Table 1**) (63). They also observed that circRNA was more stable than linear RNA and could localize to cytoplasm and nucleus. A recently developed platform used an engineered multifunctional circRNA, circSC25-αPD1, which acts as a sponge for oncogenic miR-25 and expresses an anti-PD-1 scFv (single-chain variable fragment), aiming to simultaneously disrupt oncogenic pathways and enhance the immune response against HCC (**Figure 3**, **Table 1**) (65). This engineered circRNA demonstrated strong tumor-suppressive effects in both *in vitro* cellular assays and *in vivo* mouse models, combining miRNA sequestration and protein translation functions in a single platform (65). Systemic toxicity was also evaluated, and no significant toxic side effects were observed. Another study developed a novel approach by synthesizing circDNA and circRNA with miRNA sponge-like functions for several oncogenic miRNAs that target different tumor suppressor genes, such as *ANGPTL1* (Angiopoietin Like 1), *SOCS3* (Suppressor Of Cytokine Signaling 3), and *EHHADH* (Enoyl-CoA Hydratase And 3-Hydroxyacyl CoA Dehydrogenase). Their rescue significantly inhibited malignant progression of HCC in both *in vitro* and *in vivo* mouse models (**Table 1**) (64).

## 5 Emerging circRNA-based therapeutic approaches

Insight into circRNA-based therapies for HCC reveals a range of promising approaches and a clear opportunity for further development. Although various therapies utilizing circRNA technologies are actively being developed and often target one or several factors, they have not yet been applied to HCC. Nevertheless, we reviewed these approaches to learn from their discoveries and potentially draw inspiration to advance the development of circRNA therapies for HCC and other cancers.

One emerging technology for cancer treatment is the production of oncolytic circRNAs. By expressing engineered GSDMD (gasdermin D) circRNA, which targets and interacts with the mitochondrial inner membrane cardiolipin, Feng et al. induced mitophagy and subsequently demonstrated reduced tumor growth rates and improved response to T lymphocytes in adenocarcinoma (142). While the off-target effects of this method, due to its broad cancer specificity, remain an issue before clinical trials, this same specificity also offers the advantage of potential application to a wider range of cancers.

Another unique approach, still in early clinical stages for some cancers, is targeted protein degradation. PROTACs (proteolysis-targeting chimeras) first entered clinical trials in 2019 (143), are a class of synthetic heterobifunctional molecules that link a ligand targeting a specific protein with another ligand that recruits and binds an E3 ubiquitin ligase. Simultaneous binding induces ubiquitination of the targeted protein by the ubiquitin-proteasome system (144). The effectiveness of this principle has been successfully demonstrated in multiple phase II clinical trials, for example, in the treatment of prostate and breast cancer by targeting and degrading androgen receptors (143). Although still at the preclinical stage, several targets for the treatment of HCC, such as CBP (CREB binding protein) (145) and SIRT6 (Sirtuin 6), which are involved in oncogenic gene regulation (146), have been successfully degraded *in vitro* or *in vivo* in mouse models, resulting in reduced tumor progression. Standard PROTAC platforms require non-covalently binding ligands to enable recycling of PROTACs for prolonged effect, which limits target selection and causes toxicity due to the concentration required to achieve the desired effect. Using circRNA platforms to continuously generate PROTACs could reduce toxicity and other quantity-related issues, such as off-target effects, while also loosening restrictions on ligand design and allowing for the development of more viable therapeutics. In this case, the circRNA platform would be delivered to the cell and translated into fully functional, mass-produced PROTACs, including their own linked E3 ligase, reducing the need for host cell ligase utilization. In this way, target proteins such as tumor promoters could be selectively degraded using the host proteasome pathway. It is noteworthy that there are ongoing attempts to express PROTACs from synthesized circularized mRNA to avoid the drawbacks of non-circRNA PROTACs; however, no papers demonstrating this principle have yet been officially published (147). The design of molecularly stable PROTAC linkers - and with that more precise and functional PROTACs - already present a challenge when constructing regular PROTACs *in vitro*, where the process can be more closely controlled (148). Therefore, attempting to express a fully stable and functional PROTAC molecule directly from a single circRNA will most likely - just as with regular PROTACs - require accurate *in silico* predictions before achieving the desired stability (149).

Regarding circRNA-protein interactions, sequestration of RBPs is not the only available mechanism. The ability of circRNAs to bind multiple proteins enables them to function as scaffolds and modulate process efficiency. In such cases, circRNAs could fulfil multiple roles simultaneously such as protein or RNA binding, producing certain intermediates and spatially aligning them in proximity. Multiple naturally occurring interactions of circRNAs with the HuR protein have already been demonstrated and linked to HCC (44–46). Such circRNAs could potentially serve as inspiration for protein scaffold designs targeting the oncogenic HuR. The use of circRNAs as scaffolds for multistep pathways remains largely unexplored but holds significant potential, although it is still speculative.

Currently, emerging methods lack sufficient translational evidence, even in successfully published studies, and face significant unresolved barriers before clinical approval. Studies on their molecular stability, off-target effects, and optimal delivery mechanisms are also lacking. However, we believe that highlighting these potential uses would benefit future considerations of novel designs, including those for HCC therapies.

## 6 Delivery systems

These therapeutic approaches remain under consideration at this stage, and as more potential therapeutic targets emerge, the landscape will inevitably evolve. Therefore, the most significant contributions to the broader field of circRNA-based therapies may arise from advancements in method optimization, including delivery vectors, improved IRES sites and other backbone optimizations, circRNA synthesis, purification methods, and their application in standardizing circRNA platform designs (147). For most platforms, LNPs have been the preferred delivery mechanism, sometimes with therapy-specific modifications such as immunomodulatory LNPs or shielding nanoparticles coupled with the circRNA cargo. While LNP optimization has clear benefits, drawbacks include limited transgene expression due to unreliable nuclear trafficking, toxicity, instability, and the unresolved innate cellular immune response to ectopic nucleic acids (150). However, a standardized LNP vector design for cancer therapies has not yet been established. Viral vectors are also in use, such as AAV (adeno-associated virus) and lentiviral vectors used in CAR-T therapies (93, 151). Both carry risks of genome integration, require repeated dosing due to low immunogenicity, and have a strict genomic length limit of less than 4.7 kb due to capsid packaging. Nevertheless, AAV vectors are approved for clinical use and could be considered for smaller circRNAs, such as those with miRNA sequestration functions. Another vector to consider is artificial exosomes, which, due to their larger cargo capacity, canonical membrane fusion mechanisms, and general host acceptance (152, 153), could reduce toxicity and increase specificity compared to LNPs. Studies have successfully designed artificial exosomes and addressed the challenge of packaging circRNA by introducing fused exosomal protein CD63-HuR (154). Exosomes as a delivery method, likely due to their relatively early stage of development, also face issues with nuclear trafficking, lack of large-scale extraction and purification methods, quality control, and overall delivery efficiency (155–157). However, their large cargo capacity and good biocompatibility theoretically allow for greater flexibility in cargo additions and design.

A relatively simple method with low immunogenicity is electroporation and its various forms. By generating short electrical pulses, temporary membrane pores are created, allowing direct physical transfer of circRNA into the target cell. This bypasses most biological transfer barriers, such as cargo size limitations or the inability of innate cellular mechanisms to uptake the material. Although electroporation still has an upper cargo size limit, it is relatively larger than that of other delivery systems, such as viral transfections. This approach is sometimes used for various CAR-T or TCR-T therapies, including circRNA-based TCR-T therapy (111) due to its relatively good transfection efficiency and well-established methodology. However, it does have critical drawbacks, mainly cell damage and major biological disruptions (158).

Several key limitations remain prevalent in circRNA-based therapeutic approaches. These limitations slow the development of therapeutics and delay their entry into clinical trials. The scalability of both delivery vectors and their cargo remain low, making therapies expensive, especially with more complex methods such as exosomes and viral vectors. The varied and often high immunogenicity of methods such as viral vectors and LNPs poses challenges due to different immune responses. These can cause unwanted effects such as toxicity and can introduce complex variables when studying these methods. Additionally, all currently available platforms still have limited transfection efficacy and would benefit from further research in this field (147).

## 7 Remaining considerations and future developments

A major overarching issue in circRNA studies, in addition to the inefficient synthesis and purification methods discussed elsewhere (147), is that less than 20% of human circRNAs overlap with those in the mouse models most commonly used in research. This raises questions about several results and our understanding of tumor microenvironments. The processes occurring within the human TME (tumor microenvironment), including HCC, are complex, complicating studies of interactions between the host immune system and tumors. Mouse cancer models are crucial for our basic understanding of human tumor biology and response to therapy, but they often do not accurately recapitulate basic tumor biology, as is evident when comparing outcomes with clinical studies. Different types of organoids and patient-derived tumor organoids can be used to study the TME but lack the physiological complexity of *in vivo* systems (159). This has led to growing interest in research using humanized mice, which are immunodeficient mice co-engrafted with human tumors and key components of the immune system (160, 161). Modern humanized mouse models provide an improved *in vivo* method for studying tumors due to their TME and interactions with the host immune response, including specific interactions between engrafted human tumors and immune components. It is also possible to study the development and survival of human innate immune populations transplanted into humanized mice, as well mice engrafted with matched patient tumors and immune cells (162, 163). However, humanized mouse models still face limitations, including restricted development of mature innate immune cells and limited ability to generate antigen-specific antibody responses. Given the complexity of generating humanized mice for experimental studies, the advantages and limitations of each specific model need to be carefully considered. So far, few studies of circRNA have been conducted on humanized mice (11, 164), most likely because the circRNA expression profiles of mice and humans are tissue-specific and differ from each other (165). The introduction of humanized mouse models (166) and more advanced 3D models brings us closer to a realistic model of HCC. Advanced 3D models such as assembloids, organoids, and tumors-on-chip better simulate the functions and architectures of *in vivo* organs and TME through precise tissue organization. Signals from the ECM are also fundamental for cancer development and progression and cannot be studied in 2D models (167, 168). Further development of these models and studies of circRNA expression will most likely be needed for a proper understanding of cancer dynamics and circRNA functionality, whether as biomarkers or therapeutics.

Currently, there is no dedicated regulatory guidance for RNA-based medicines, and no consensus definition of RNA medicines from a regulatory perspective. RNA-based medicines are assigned to the regulatory framework for chemicals or biologicals depending on their properties and production (169). For example, for RNA-based vaccines, vaccine development guidelines are recommended, while ASO, miRNA, and siRNA are categorized as chemicals. Additionally, the categorization of some RNA-based medicines differs between the FDA (Food and Drug Administration) and the EMA (European Medicines Agency) (169). In 2023, the EMA organized a conference to promote discussion on regulatory and scientific issues related to emerging RNA technologies (170). CircRNA-based therapies share the same regulatory issues as other RNA-based therapies.

Off-target effects remain a significant challenge in RNA therapeutics. These effects occur when therapeutic RNA molecules interact with genes other than their intended targets, potentially causing safety concerns. In RNAi-based therapies, including circRNA-based miRNA sequestration, off-target effects mainly results from partial complementarity between therapeutic RNAs and unintended mRNA targets (171). Even short, limited complementarity within the seed region of RNAi molecules can induce unintended gene silencing. Chemical modifications or CRISPR-Cas13 editing of RNA molecules, along with careful RNA sequence design using precise bioinformatics analysis, can minimize potential off-target binding sites (172).

Innate immune activation often occurs with RNA therapeutics due to their inherent immunogenicity. While this immunostimulatory property may be advantageous for RNA vaccines, it may reduce efficacy and raise safety concerns in other RNA therapeutics. CircRNA is less immunogenic and mutagenic, making it safer than conventional mRNA vectors. This makes circRNA platforms an attractive option for developing the next generation of RNA-based therapies. Additionally, the immunogenicity of circRNAs can be further suppressed, if needed, through *N*6-methyladenosine (m^6^A) modification (173). This modification renders RNA unrecognizable to the RIG-I (RNA pattern recognition receptor), which is part of the cascade responsible for detecting foreign RNA and triggering an immune response. The delivery systems used for RNA therapeutics also influence their immunogenicity profile. LNPs, as the primary example, have been noted to potentially elicit immune responses themselves. Optimizing the composition and physicochemical properties of nanoparticles can help minimize immunogenicity (174), offering prolonged expression with fewer drawbacks.

Combining multiple therapies has emerged as a promising approach to overcome limitations such the low immunogenicity of individual therapies. When treating complex diseases that often develop resistance to therapies, combining therapeutic strategies can be invaluable. This approach involves using multiple RNA therapeutics or integrating RNA therapeutics with conventional drugs and treatment modalities (172). The primary advantage of combining therapies is in their ability to simultaneously target multiple pathways and mechanisms. In this way, a greater and more efficient immune response can be achieved, reducing toxicity and other negative consequences arising from repeated therapeutic deliveries. Different therapeutic RNA molecules can be combined with each other or with traditional chemotherapeutic agents for more comprehensive and effective treatment strategies (175, 176). Some studies have further advanced this approach by combining immune checkpoint inhibitors with RNA therapeutics. Advanced nanoparticle systems, such as LNPs and polymeric nanocarriers, can be engineered to encapsulate and simultaneously deliver multiple RNA molecules or combinations of RNA and small-molecule drugs (177). This approach can also be used to enhance therapeutic efficacy of circRNA-based therapies.

## 8 Conclusions

CircRNAs are covalently closed RNAs whose expression varies across tissues and disease states. Their high stability and multiple endogenous molecular functions make them promising biomarkers and components of RNA-based therapeutics. Their functions include acting as miRNA sponges, regulating transcription, splicing, translation, and post-translation, as well as being translated into proteins. They can bind various RNAs and proteins and are known cargo of exosomes, thereby affecting neighboring tissue. Their broad molecular functions enable their use in diverse therapeutic applications, many of which have shown preclinical success. CircRNAs can be used for the expression of therapeutic proteins with high stability and low immunogenicity: (a) in RNA vaccines to encode tumor neoantigens or TAAs to stimulate immune responses; (b) in CAR-T and TCR-T therapies to express different CARs and TCRs in T cells; and (c) for intratumoral expression of cytokines to modulate the tumor microenvironment. CircRNAs can also act as sponges, sequestering oncogenic RNAs and proteins in tumors. Furthermore, several emerging approaches should be considered, such as (a) oncolytic circRNAs, which induce autophagy and activate the immune system; (b) PROTACs via circRNA, with potential for targeted protein degradation; and (c) use as circRNA scaffolds to modulate molecular processes. Delivery systems are also being developed, ranging from lipid nanoparticles to artificial exosomes. Most applications have shown success in preclinical *in vitro* and *in vivo* studies. Some circRNA-based therapies, such as CAR-T via circRNA, have entered clinical trials for non-HCC cancers. However, no HCC-specific circRNA-based therapies have yet reached the clinical trial phase, and most remain in preclinical development. Examples include a circRNA vaccine expressing a mutated *PTPN2* neoantigen and circRNA sponges for various oncogenic miRNAs such as miR-25, etc.

CircRNA-based therapies face the same limitations and considerations as other RNA-based therapies. They offer advantages over other RNA-based therapies, such as longer expression and greater stability. However, at this stage, we cannot fully predict all the drawbacks of using circular RNA. Their greatest strength is their broader versatility compared to other RNA-based therapies. Studies of endogenous circRNA functions in tumor cells have already revealed a wide range of targets, molecular mechanisms, and effects on tumors. As single molecules, they serve in multiple combined roles, affect more targets and processes to reduce tumor growth, activate the immune response, and even provide prophylactic effects. However, several challenges hinder clinical translation: First, the lack of standardized expression platforms and delivery systems prolongs studies due to novel variables introduced by specific therapeutic designs. Standardized backbones with optimized IRES sites, modulated immunogenicity through m6A modifications, and efficient backbone construction would help accelerate therapies to clinical trials. Similarly, delivery vector designs and their efficiency still vary among different circRNA therapies. For example, lipid nanoparticles are often used because they are easy to mass-produce, cost-effective, and already approved for clinical use, but they have drawbacks such as low transfection rates. Exosomes are a promising novel delivery vector, but more research is needed before they can be mass-produced and integrated into clinical studies. Another major hurdle for clinical translation is the limited overlap between human and mouse circRNAs. This highlights the need for better tumor models, as current studies do not accurately represent circRNA cancer dynamics. Humanized mouse models with altered immune systems or 3D tumor models, such as advanced organoids that more closely resemble the tumor microenvironment and extracellular matrix signaling, are likely better alternatives. Finally, regulatory guidance and definitions for RNA-based medicines are still under debate. These therapies are assigned to the regulatory framework for chemicals or biologicals depending on their properties and production methods. This distinction is often unclear, as with RNA vaccines, and classification differs between agencies such as the FDA and EMA. Nevertheless, circRNAs remain promising candidates for enabling safer, faster, longer-lasting, and more effective immunotherapies.
